# Redundant contribution of a Transient Receptor Potential cation channel Member 1 exon 11 single nucleotide polymorphism to equine congenital stationary night blindness

**DOI:** 10.1186/s12917-016-0745-1

**Published:** 2016-06-21

**Authors:** Michelle L. Scott, Emily E. John, Rebecca R. Bellone, John C. H. Ching, Matthew E. Loewen, Lynne S. Sandmeyer, Bruce H. Grahn, George W. Forsyth

**Affiliations:** Veterinary Biomedical Sciences, Western College of Veterinary Medicine, University of Saskatchewan, Saskatoon, SK S7N 5B4 Canada; Department of Biology, University of Tampa, Tampa, FL 33606 USA; Small Animal Clinical Sciences, Western College of Veterinary Medicine, University of Saskatchewan, Saskatoon, Canada

**Keywords:** Appaloosa, Congenital stationary night blindness, Transient Receptor Potential cation channel Member 1

## Abstract

**Background:**

Congenital stationary night-blindness (CSNB) is a recessive autosomal defect in low-light vision in Appaloosa and other horse breeds. This condition has been mapped by linkage analysis to a gene coding for the Transient Receptor Potential cation channel Member 1 (*TRPM1*). TRPM1 is normally expressed in the ON-bipolar cells of the inner nuclear layer of the retina. Down-regulation of TRPM1 expression in CSNB results from a transposon-like insertion in intron 1 of the *TRPM1* gene. Stop transcription signals in this transposon significantly reduce TRPM1 primary transcript levels in CSNB horses. This study describes additional contributions by a second mutation of the TRPM1 gene, the ECA1 108,249,293 C > T SNP, to down-regulation of transcription of the TRPM1 gene in night-blind horses. This *TRPM1* SNP introduces a consensus binding site for neuro-oncological ventral antigen 1 (Nova-1) protein in the primary transcript. Nova-1 binding disrupts normal splicing signals, producing unstable, non-functional mRNA transcripts.

**Results:**

Retinal bipolar cells express both TRPM1 and Nova-1 proteins. In vitro addition of Nova-1 protein retards electrophoretic migration of TRPM1 RNA containing the ECA1 108,249,293 C > T SNP. Up-regulating Nova-1 expression in primary cultures of choroidal melanocytes carrying the intron 11 SNP caused an average log 2-fold reduction of ~6 (64-fold) of TRPM1 mRNA expression.

**Conclusions:**

These finding suggest that the equine TRPM1 SNP can act independently to reduce survival of TRPM1 mRNA escaping the intron 1 transcriptional stop signals in CSNB horses. Coexistence and co-inheritance of two independent TRPM1 mutations across 1000 equine generations suggests a selective advantage for the apparently deleterious CSNB trait.

## Background

Reduced expression of the transient receptor potential cation channel, subfamily M, member 1 (*TRPM1*) in retinal ON bipolar cells causes congenital stationary night-blindness (CSNB) in Appaloosa, Knabstrupper and other horse breeds. We have reported an 1800-fold reduction of *TRPM1* mRNA expression in CSNB retinal tissue [1]. ON bipolar cells lacking the *TRPM1* cation channel are unable to depolarize in response to mGlu R6 receptor interaction with its coupled Gαo protein, interrupting signal transmission to the optic nerve [2, 3]. This failure of depolarization eliminates the b wave in the electroretinogram, providing a distinctive clinical signature for impaired night-vision in affected horses. Inherited defects in *TRPM1* channel expression or function produce this CSNB phenotype.

TRPM1 protein is also expressed in equine skin melanocytes where it participates in melanin production [4]. However, expression of *TRPM1* mRNA is only reduced ~300 fold in non-pigmented skin from CSNB horses. The coat pattern spotting of heterozygotes and the “snow cap” phenotype of homozygous CSNB horses typified in the Appaloosa breed appears to have some connection to the reduced *TRPM1* expression in CSNB skin [1]. The distinctive spotted coat pattern characteristic of heterozygotes with a single functional TRPM1 gene accounts for the selective breeding for reduced *TRPM1* expression. But the homozygous *TRPM1*-negative Snowcap stallion guaranteed to produce spotted offspring on a solid Bay mare is night-blind [5]. Interest in the molecular basis for tissue-specific effects of the equine *TRPM1* gene is enhanced by their different modes of inheritance and the divergent tissue phenotypes attending heterozygous and homozygous *TRPM1* mutations.

Sequencing the coding and flanking regions of the *TRPM1* gene did not identify a credible cause for reduced levels of transcript in CSNB horses [6, 7]. However, analysis of the CSNB retinal transcriptome revealed the transposon-like insertion of a retroviral LTR in intron 1 of the *TRPM1* gene [8]. This insertion introduces multiple polyadenylation signals likely to prematurely truncate the primary CSNB TRPM1 transcript. This finding accounts for decreased TRPM1 expression, but fails to explain tissue-specific differences (retina vs skin) in the extent of reduced TRPM1 expression in homozygous CSNB horses or in tissue specificity for the dominance pattern of the retinal and skin phenotypes of the TRPM1 mutations. Partial dominance of the Leopard coat pattern spotting phenotype, but recessive inheritance of night blindness could be connected to tissue-specific differences in TRPM1 mRNA expression or tissue processing.

Previous to discovery of the retroviral LTR insertion in intron 1 of the *TRPM1* gene we had identified an intronic mutation associated with CSNB and coat pattern phenotypes. This ECA1 108,249,293 C > T SNP located in intron 11 of the TRPM1 transcript [6] introduces a binding site for the tissue-specific neuro-oncological ventral antigen 1 (Nova-1). Nova-1 protein is a splice enhancer which can reduce the survival of transcripts carrying its recognition motifs within their RNA sequence [9]. Nova-1 is expressed at highest levels in neural tissue such as retina [10] where TRPM1 expression is particularly affected in the CSNB condition. However, there is much less Nova-1 expression in skin; providing a basis for tissue-specific differences in survival of the TRPM1 transcript carrying the intron 11 SNP.

This manuscript reports in vitro and in situ Nova-1 protein interactions with, and effects on the survival of mutant mRNA carrying the *TRPM1* intron 11 C > T SNP.

## Methods

### Primary choroidal melanocyte cultures

Retinal and skin tissue samples were obtained from horses humanely euthanized for other purposes at the Veterinary Medical Center of the University of Saskatchewan following the Canadian Council on Animal Care Guidelines for Experimental Animal Use and approved by the University of Saskatchewan Animal Care Committee. Samples for choroidal cell culture, for immunohistochemistry, and retinal and skin samples for RNA isolation were collected from an unaffected (homozygous WT) Appaloosa horse. Tissue was also collected from a CSNB-affected (homozygous for the *TRPM1* intron 11 SNP) horse for RNA isolation and choroidal cell culture. Collected choroidal cells were placed in cell culture media (DMEM media containing 10 % fetal bovine serum, 2 mM L-glutamine, 0.1 mM 3-isobutyl-1-methylxanthine, 16.2 nM phorbol 12-myristate 13-acetate, and 50 μg/mL and 50 units/mL of Penicilllin-Streptomycin) in six-well plates. Cells were incubated in 5 % CO_2_ at 37 °C. Upon reaching 90–100 % confluency (7–10 days) cells were trypsinized and passaged into 75 cm^2^ flasks. Cells from passage number 5 (p5) were used in all comparisons between the two genotypes of choroidal melanocytes.

### Transfection constructs

Nova 1 expression in cultured choroidal melanocytes was increased by transfecting cells with human Nova-1 in Origene’s pCMV6-XL5 expression vector. Microphthalmia-associated transcription factor, subtype M (MITF-M) expression was up-regulated by transfecting with an expression plasmid containing human MITF-M cDNA under control of a cytomegaloviral (CMV) promoter (a gift of Dr. Shigeki Shibahara of Tohoku University School of Medicine, Sendai, Japan) [11].

### Transfections

P5 choroidal melanocytes from a normal and a night blind Appaloosa horse were plated at a density of 6 × 10^6^ cells per flask and incubated 24 h. Media was exchanged 2 h before transfecting with 30 μg of plasmid DNA (Nova-1 pCMV6-XL5 cDNA or MITF-M pRc/CMV). Plasmid DNA solutions in 2.25 mL 2x BES buffer were mixed with 225 ul saturated CaPO_4_, incubated exactly 20 min at 20 °C and added dropwise to recipient cells [12] to produce three biological replicates for each transfection condition. Transfected cells were incubated overnight prior to RNA isolation, cDNA synthesis and q-PCR.

### q-PCR

RNA was isolated from tissues and cultured cells using the Trizol protocol and treated with the Turbo DNase according to manufacturer’s recommended procedure (Invitrogen). RNA quantity and quality were tested for A260/280 absorbance and for size (Agilent 2100 bioanalyser). First strand cDNA synthesis was carried out with ThermoScript reverse transcriptase (Invitrogen) according to the manufacturer’s protocol. Intron-spanning primer pairs specific for TRPM1, Nova-1, MITF-M and β-actin were designed and tested for efficiency and for production of respective unique products (Table 1). qPCR was carried out on an Agilent Mx3005P instrument using SYBR Green for product detection. Relative quantification (RQ) was calculated using 2^-ΔΔCT^ method [13] on 6 replicated RT reactions for each condition. qRT-PCR data were analyzed according Schmittgen and Livak [14].

**Table 1:** PCR primers used to amplify human (H s) and equine (E c) cDNA templates

Primer	5′ – 3′ sequence	Product bp	Efficiency
E c Nova-1 F16	CAA ACT ACC ACC AAG TCC TC		
E c Nova-1 R224	CTC ACA GTG ACA ACC CTC TC	209	110 %
E c Nova-1 F1024	GGG ACA TTT GCA TTA GGT AG		
E c Nova-1 R1454	ATG GGG TAA AGG AGG GGT TA	431	
H s Nova-1 F310	ACA TTG CCA TCT TCC CCA AC		
H s Nova 1 R371	TGG AGG TGG TCA TGG GAT CA	62	
E c MITF-M F 408	CTT GAT GGA TCC TGC CTT G		
E c MITF-M R573	GGG AAA AAT ACA CGC TGT GA	166	110 %
E c βactin F55	GCC GTC TTC CCC TCC AT		
E c βactin R135	GCC C AC GTA TGA GTC CTT CTG	81	121 %
E c TRPM1 F2254	GAC GAC ATC TCC CAG GAT CT		
E c TRPM1 R2326	TGC TCG TCG TGC TTA TAG GA	73	113 %

### Immunohistochemistry

Immunohistochemical staining was conducted at Prairie Diagnostic Services, Saskatoon, SK on an automated staining platform (Code-On Histomatic Stainer, Fisher Scientific, Edmonton, AB, Canada). Heat-induced epitope retrieval was performed (sodium citrate buffer, pH 6.1) and primary goat polyclonal antibody (goat anti-Nova-1, Santa Cruz Biotechnology Inc, Dallas, TX) was used at dilutions of 1:10 and 1:20. Primary antibody-dependent binding of rabbit anti-goat immunoglobulins was detected with an avidin-biotin immunoperoxidase complex reagent (Vector Labs; Burlingame, CA), with 3,3′-diaminobenzidine tetrahydrochloride (Electron Microscopy Science, Ft. Washington, PA) as chromogen.

### Gel shift assay

Custom RNA oligomers corresponding to 60 bases spanning the *TRPM1* intron 11 SNP, or to the positive control RNA used in Nova-1 binding protocol (Table 2) were purchased from GE Healthcare Dharmacon. 10 ng of each RNA oligomer was denatured at 80 °C for 5 min, cooled to room temperature, then incubated for 30 min @ 20° with 500 ng of wheat germ recombinant human Nova-1 protein (Abcam, Toronto, ON Canada) in buffer containing 0.5 M LiCl, 1 mM MgCl_2_, 20 mM Tris–HCl pH 7.6, and 20 ng of yeast tRNA per reaction. Samples were electrophoresed in TBE running buffer on non-denaturing Tris-glycine-buffered 8 % polyacrylamide gels at 75 V for 1.5 h. Four replicate gels were stained in SYBR gold for 35 min, then imaged for 8 s exposures. Pixel intensity of fluorescent bands was quantitated with Image Pro Plus 7.0 software by Media Cybernetics. GraphPad Prism software was used for unpaired *T*-test analyses of output data.

**Table 2:**
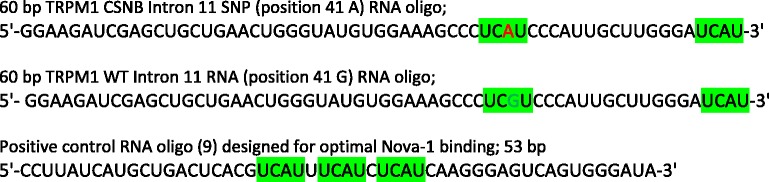
RNA oligomer sequences tested in gel shift assay for interaction with Nova-1 protein. Highlighted –UCAU- motifs are predicted to interact with the Nova-1 protein

## Results

**Fig. 1:**
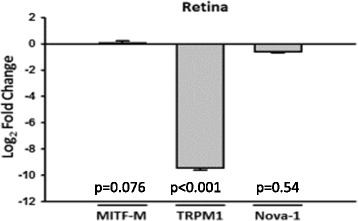
mRNA expression in night-blind equine retina. Relative expression of mRNA coding for the three proteins of interest from retina of a night blind (CSNB) horse, compared to expression in a control horse with normal night vision. Triplicate qrt-pcr reactions were carried out on single RNA samples

Differences in expression of TRPM1 mRNA reported previously between retinal tissues from CSNB and control horses [1] were confirmed in tissues sourced for this study. TRPM1 mRNA expression (normalized to β-actin) in retinal tissue from the ERG-confirmed night-blind (CSNB) horse used in this study was 0.26 ± .022 % of the expression level measured in retina of a control horse (Fig. 1).

Expression of mRNA coding for the choroidal melanocyte marker protein (and TRPM1 transcription factor) MITF-M [15, 16], was similar in WT and CSNB retinal tissue and also between WT and CSNB cultured melanocytes.

**Fig. 2:**
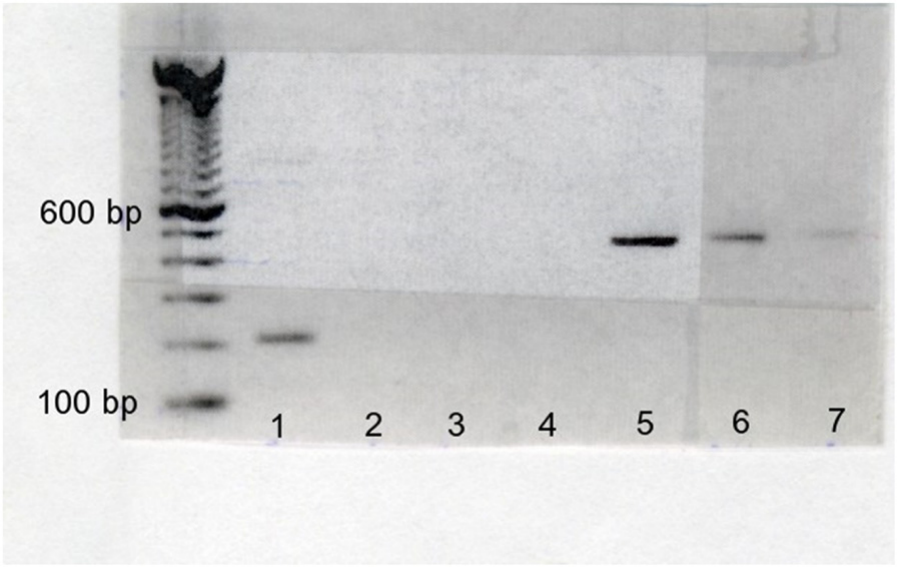
Tissue-specific Nova-1 expression. RNA from retina (lanes 1 & 5), pigmented (lanes 2 & 6) and unpigmented skin (lanes 3 & 7) from a normal horse was reverse-transcribed using equal amounts of cDNA template in 30 cycles of PCR. PCR products were separated on agarose gel electrophoresis and identified by fluorescence with ethidium bromide. Lanes 1–3 were amplified with intron-spanning primers E c Nova-1 F16 – R224, product length = 209 bp. Lanes 5–7 were amplified with E c Nova-1 F1024 – R1454 primers spanning the stop codon, ;product length = 431 bp

Buckanovich et al. reported that Nova-1 expression is confined to tissues of neural origin [9]. In this study Nova 1 cDNA template was identified in retinal RNA by agarose gel electrophoresis (Fig. 2. lane 1) but was not detectable after 30 pcr cycles with intron-spanning primers with equal amounts of cDNA template prepared from equine skin RNA (lane 2). PCR amplification from the same cDNA template with a primer pair spanning the stop codon revealed DNA contamination of the cDNA template made from RNA treated with DNase. This difference in Nova 1 mRNA expression between retina and skin shown in Fig. 2 has been confirmed by qrtPCR (E.J. data not shown).

**Fig. 3:**
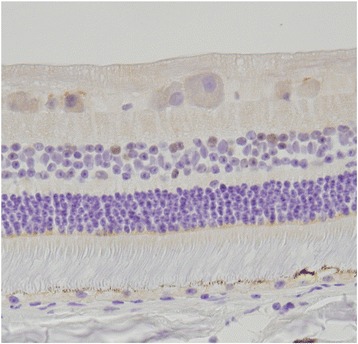
Nova-1 expression in equine retina. Immunohistochemical identification of Nova-1 in equine retinal pigment epithelial cells and in bipolar cells in the inner nuclear layer. Orientation of retinal cross-section from lower to upper region is as follows: Choroid, retinal pigment epithelia, rod and cone photoreceptors, outer nuclear layer, inner nuclear layer (containing the ON-bipolar cells), ganglionic cell layer, inner limiting membrane

Nova-1 expression must co-localize with TRPM1 in the retinal ON-bipolar cell in order for the Nova-1 binding site created by the TRPM1 intron 11 SNP to affect processing/stability of TRPM1 transcripts. Immunohistochemistry with goat anti-equine Nova-1 antibody shows the presence of Nova-1 antigen in a subpopulation of bipolar cells in the inner nuclear layer of equine retina (Fig. 3).

**Fig. 4:**
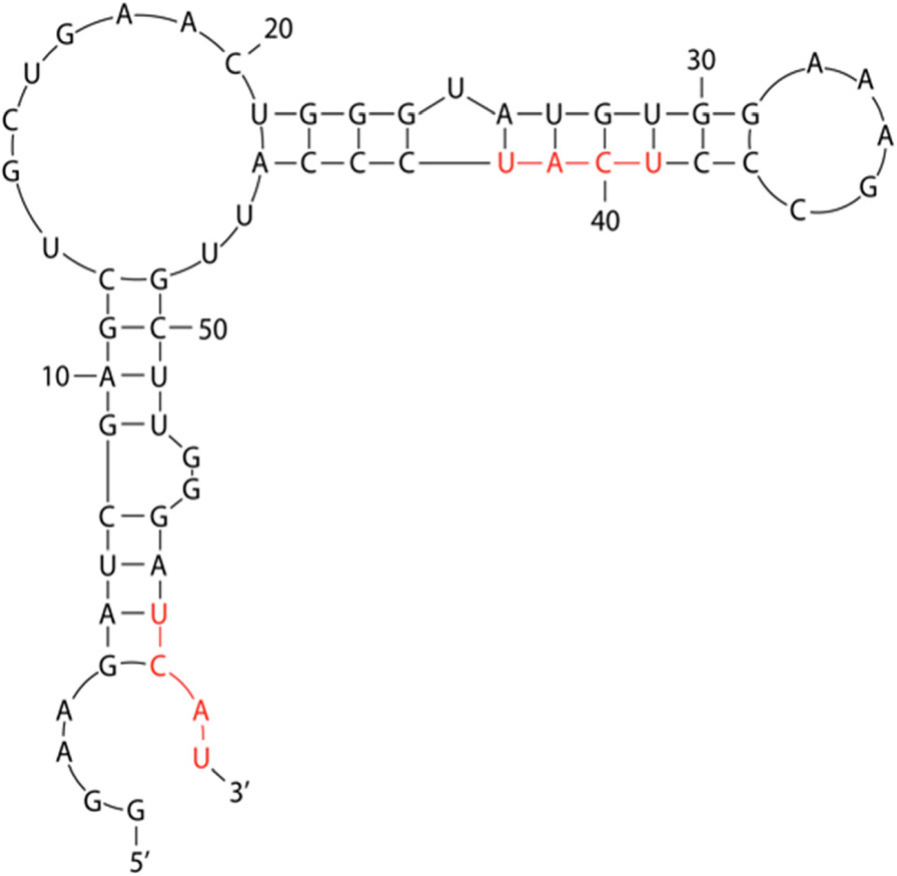
M-fold minimal energy model of RNA secondary structure. Predicted two-dimensional secondary structure with base-pairing of a synthetic 60-mer transcript spanning the ECA1 108,249,293 C > T TRPM1 intron 11 SNP. Transcription of the SNP produces the adenine residue at position 41 in the RNA oligomer

Nova-1 is reported to prefer binding to RNA containing 2 or 3 repeats of the -UCAU- recognition sequence motif [10]. Nova-1 binding may also be facilitated by secondary RNA structure where 5′-UCAU-3′ motifs occur in the predicted loop of stem-loop structures. M-fold software predicts that the ECA1 108,249,293 C > T SNP will lie close to the tip of a loop of a predicted secondary “hairpin” structure in the primary TRPM1 transcript (Fig. 4). The -UCAU- motif produced by the SNP is in an exposed location (position 39 in the 60-mer) which is likely to be stabilized by extensive complementary base pairing in the stem region of the oligomer.

The other consensus Nova-1 site at position 57 in the 60-mer occurs in the presumably less accessible stem of the hairpin in the native WT transcript.

**Fig. 5:**
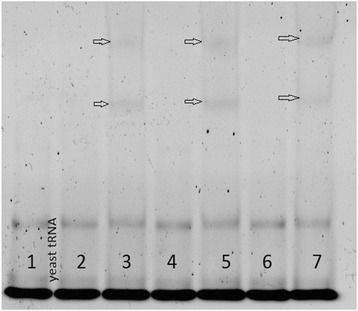
The effect of the presence of Nova-1 protein on electrophoretic migration of synthetic RNA oligomers. RNA bands were visualized after electrophoresis by SYBR-gold fluorescence. Lane 1; Background yeast tRNA only. Lane 2; TRPM1 WT intron 11 60-mer RNA oligo. Lane 3; TRPM1 WT intron 11 60-mer RNA oligo plus Nova-1 protein. Lane 4; TRPM1 CSNB Intron 11 SNP 60mer RNA oligo. Lane 5; TRPM1 CSNB Intron 11 SNP 60mer RNA oligo plus Nova-1. Lane 6; positive control 60-mer oligo containing 3 UCAU motifs. Lane 7; positive control 60-mer oligo plus Nova-1. Location of RNA-Nova-1 complex with retarded mobility is indicated by open arrows

The gel-shift assay result shown in Fig. 5 was used to test the three synthetic RNA oligonucleotides described in Table 2 for possible in vitro interactions with Nova-1 protein. Small 53-60-mer synthetic RNA oligomers migrated more rapidly than the yeast tRNA (73–93 nucleotides) used to prevent non-specific RNA-protein binding to Nova-1, and were lost from the bottom of each lane prior to staining. The excess yeast tRNA appears as the intensely-stained band at the bottom of each lane.

Preincubation of Nova 1 protein with a 53 base “positive control” RNA oligomer containing three tandem repeats of UCAU recognition sequence (Fig. 5 lane 7) formed a complex which retarded migration of this oligomer in polyacrylamide media under the non-denaturing conditions used for electrophoresis. Even a single occurrence of the UCAU motif in the WT TRPM1 oligomer (lane 3) was apparently sufficient to allow Nova-1-dependent-RNA complex formation and retarded electrophoretic migration.

Quantitative pixel intensity measurements of the CYBR Gold fluorescence products in Fig. 5 showed a 154 ± 34 % increase in fluorescence intensity for the retarded positive control RNA bands containing 3 repeats of the Nova-1 binding motif (lane 7) in four replicates of this gel-shift assay compared to the native TRPM1 oligomer bands (lane 3) containing a single copy of the motif. Insertion of a second –UCAU- Nova-1 target motif corresponding to the transcript of the intron 11 SNP into the pyrimidine-rich area of the 60-mer increased the fluorescence intensity of the Nova-1- RNA complex in lane 5 to 157 ± 17 % of that of the native TRPM1 oligomer in the 4 gel-shift replicates. Limits to precise quantitation in this assay make the extent of Nova-1 binding to the 60-mer TRPM1 oligo containing the CSNB SNP indistinguishable from Nova-1 binding to the positive control 53-mer RNA of Buckanovich and Darnell [10] identified in Table 1.

**Fig. 6:**
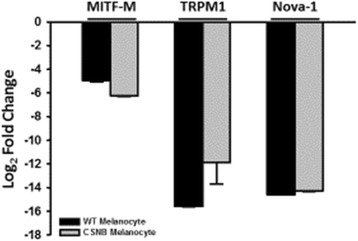
Reduced expression of tissue-specific choroidal melanocyte marker genes with cell passage. ΔΔCT values compare mRNA expression levels measured by q-RT-PCR from six separate flasks of each genotype of melanocytes at the 5th passage to fresh retinal tissue relative to β-actin

Evidence for the presence of Nova-1 in retinal inner nuclear bipolar cells and for physical interactions of Nova-1 protein with TRPM1 mRNA suggests the possibility of in vivo effects of Nova-1 on TRPM1 mRNA containing the transcript of the intron 11 SNP. The cultured primary choroidal melanocytes obtained from the CSNB and from the normal horse were used to investigate this possibility. Melanocyte passage to P5 was necessary to obtain sufficient cell numbers for replicate transfections. However melanocyte mRNA expression diminished significantly (*p* < 0.001) with successive choroidal melanocyte passage for all three marker genes used in this study (Fig. 6).

An in situ cell culture model requires sufficient MITF-M protein to promote TRPM1 expression and Nova-1 protein to interact with the binding motif provided by the intron 11 SNP. Increased expression of marker mRNA in de-differentiated melanocyte cultures was obtained by transfecting P5 melanocytes singly with the vectors expressing MITF-M and Nova-1, and then with dual transfection to increase expression of both proteins. Single transfections with MITF-M increased TRPM1 expression ~4-fold in 3 biological replicate transfections of WT melanocytes and ~2-fold in CSNB melanocyte cultures (Table 3). Single transfections with Nova-1 had the opposite effect on TRPM1 mRNA expression, decreasing WT melanocyte TRPM1 expression 5-fold and decreasing CSNB melanocyte TRPM1 expression 55-fold. The combined dual transfection reduced WT melanocyte TRPM1 mRNA expression 14-fold (1/0.072) and CSNB melanocyte TRPM1 mRNA by ~150-fold (1/0.0068) (Table 3). These genotype differences in response to dual transfection were significant at the *p* < 0.001 level.

**Table 3:** Relative amount of TRPM1 mRNA detected in cultured choroidal melanocytes transfected with expression vectors coding for MITF-M and/or Nova-1 expression. *n* = 6 replicate rt q-pcr reactions

	Effect of transfection on relative TRPM1 mRNA level				
	Wild type melanocytes		LP/LP melanocytes		*P*
	ΔΔCT	Fold change	ΔΔCT	Fold change	
MITF-M	−2.1 ± 0.8	4.3	−0.8 ± 1.9	1.74	0.14
Nova-1	2.3 ± 0.8	0.20	5.8 ± 1.1	0.018	<0.001
MITF-M & Nova-1	3.8 ± 0.3	0.072	7.2 ± 0.9	0.0068	<0.001

## Discussion

Nova-1 is a neuron-specific RNA splicing factor that favors alternative exon inclusion during transcript maturation. Mutations inserting Nova-1 recognition sites into transcripts expressed in neural tissue could significantly disrupt normal transcript processing/maturation. Co-expression of Nova-1 with TRPM1 in bipolar cells in the neural retina indicates potential trouble for the mutant TRPM1 transcripts containing the novel Nova-1 binding site. However, the degree of jeopardy attending generation of a new Nova-1 binding site could be influenced by accessibility of the new site to its protein ligand. M-fold structure predictions for synthetic oligomers constructed for this study suggest that the TRPM1 intron 11 SNP lies in an exposed loop area of the nascent transcript, and that the SNP-induced G > A change to the TRPM1 transcript helps stabilize this exposed target loop by adding an extra base pair to the proposed secondary structure. This conjectured importance of a new Nova-1 binding site on the side of a modelled exposed loop is supported by the outcome of the gel shift results reported above.

The existence of a native WT Nova-1 binding site distal to the intron 11 SNP is of interest because M-fold software predicts this Nova-1 binding site to be relatively inaccessible, located at the base of a “stem” in the secondary structure. The gel shift finding of enhanced binding of the mutant CSNB oligomer containing a second site supports in vivo validity of the M-fold model as the single Nova-1 site in the stem permits the binding of less oligomer than the combined two sites corresponding to the CSNB mutant transcript. As observed by Buckanovich and Darnell [9], it is also possible that the combination of two Nova-1 sites on the SNP-containing oligomer outweighs the importance of Nova-1 accessibility to any single site.

One could speculate that greater down-regulation of TRPM1 mRNA expression in CSNB retina (~1800-fold) compared to skin (~300-fold) might be partially attributed to localized Nova-1 expression in ON-bipolar cells in the retina. However, connections of tissue differences in Nova-1 expression to the dominance of coat pattern and the recessive inheritance of night blindness are not apparent.

Transcriptome analysis unequivocally demonstrates termination of TRPM1 transcripts at polyadenylation sites in the long terminal repeat (LTR) insertion in intron 1 [8]. This study documents the existence of a second mutation that can apparently decrease the production of TRPM1 mRNA in the CSNB horse. The co-existence of these two mutations in modern horse breeds, each of which may be able to independently reduce or eliminate the TRPM1 transcript in the ON-bipolar cell of the retina, is an untoward situation. Linkage between the intron 1 LTR insertion and the intron 11 SNP has been documented to exist in equine DNA samples dating to 16,000 years BP [17]. Other than a genomic proximity of ~60 kb, there is no obvious selection pressure to preserve this linkage for over 1000 equine generations. The recent finding that some modern Noriker horses carrying the LTR insert lack the intron 11 SNP demonstrates that a crossover between the two loci does occur, and that the predicted coat pattern phenotype expression exists without the intron 11 SNP [6]. We are not aware of any documented observations of progeny carrying the other crossover product; the intron 11 SNP without the LTR insert. Such horses should exist, but may lack the coat color phenotype to attract attention? So the coat pattern and night-blindness phenotypes of the isolated intron 11 SNP remain an issue for future resolution.

## Conclusions

The C > T SNP in intron 11 of the TRPM1 transcript (ECA1 position 108,249,293) found in CSNB Appaloosa horses introduces a functional binding site for the tissue-specific neuro-oncological ventral antigen 1 (Nova-1). The electrophoretic migration of RNA oligomers containing the translation product of the SNP is retarded in the presence of Nova-1 protein. Up-regulating Nova-1 expression in cell lines containing the intron 11 SNP significantly reduces TRPM1 expression. Hence, night-blind Appaloosa horses have a second independent mutation which may contribute to reducing the amount of functional TRPM1 transcript in retinal ON-bipolar cells.

## Abbreviations

CMV, cytomegalovirus; CSNB, congenital stationary night blindness; ECA1, horse chromosome one; LTR, long terminal repeat; Nova-1, neuro-oncological ventral antigen 1; SNP, single nucleotide polymorphism; TRPM1, Transient Receptor Potential cation channel Member 1
